# Body mass index and physical activity and the risk of diverticular disease: a systematic review and meta-analysis of prospective studies

**DOI:** 10.1007/s00394-017-1443-x

**Published:** 2017-04-09

**Authors:** Dagfinn Aune, Abhijit Sen, Michael F. Leitzmann, Teresa Norat, Serena Tonstad, Lars J. Vatten

**Affiliations:** 1 Department of Epidemiology and Biostatistics, School of Public Health, Imperial College London, St. Mary’s Campus, Norfolk Place, Paddington, London, W2 1PG UK; 2 Department of Public Health and General Practice, Faculty of Medicine, Norwegian University of Science and Technology, Trondheim, Norway; 3 Bjørknes University College, Oslo, Norway; 4 Department of Epidemiology and Preventive Medicine, Regensburg University Medical Center, Regensburg, Germany; 5 Department of Preventive Cardiology, Oslo University Hospital, University of Oslo, Oslo, Norway

**Keywords:** Body mass index, Physical activity, Diverticular disease, Systematic review, Meta-analysis

## Abstract

**Purpose:**

We conducted a systematic review and meta-analysis of prospective studies of the association between body mass index (BMI) and physical activity and diverticular disease risk.

**Methods:**

PubMed and Embase databases were searched up to February 7, 2017. Summary relative risks and 95% confidence intervals (95% CIs) were calculated using a random effects model and nonlinear associations were modeled using fractional polynomial models.

**Results:**

Six cohort studies of BMI and diverticular disease risk (28,915 cases, 1,636,777 participants) and five cohort studies of physical activity and diverticular disease risk (2080 cases, 147,869 participants) were included. The summary relative risk (RR) of incident diverticular disease for a 5 unit BMI increment was 1.28 (95% CI: 1.18–1.40, *I* ^2^ = 77%, *n* = 6) for diverticular disease, 1.31 (95% CI: 1.09–1.56, *I* ^2^ = 74%, *n* = 2) for diverticulitis, and 1.20 (95% CI: 1.04–1.40, *I* ^2^ = 56%, *n* = 3) for diverticular disease complications. There was no evidence of a nonlinear association between BMI and diverticular disease risk (*p*_nonlinearity_ = 0.22), and risk increased even within the normal weight range. Compared to a BMI of 20, the summary RR for a BMI of 22.5, 25.0, 27.5, 30.0, 32.5, 35.0, 37.5, and 40.0 was 1.15 (1.07–1.23), 1.31 (1.17–1.47), 1.50 (1.31–1.71), 1.71 (1.52–1.94), 1.96 (1.77–2.18), 2.26 (2.00–2.54), 2.60 (2.11–3.21), and 3.01 (2.06–4.39), respectively. The summary RR was 0.76 (95% CI: 0.63–0.93, *I* ^2^ = 54%, *n* = 5) for high vs. low physical activity and 0.74 (95% CI: 0.57–0.97, *I* ^2^ = 39.5%, *p*_heterogeneity_ = 0.20, *n* = 2) for high vs. low vigorous physical activity.

**Conclusions:**

These results suggest that even moderate increases in BMI may increase the risk of diverticular disease as well as diverticular disease complications and that a higher level of physical activity may reduce the risk.

**Electronic supplementary material:**

The online version of this article (doi:10.1007/s00394-017-1443-x) contains supplementary material, which is available to authorized users.

## Introduction

Diverticular disease is a common disease of high-income countries and has been called a “disease of the Western civilisation” [1]. This is due to the fact that the incidence and prevalence of diverticular disease ranges more than 20- to 40-fold between high- and low-risk populations and tends to be more common in high-income countries where westernized lifestyles are prevalent [2, 3]. Secular trend studies have found that the incidence of diverticular disease has increased rapidly within countries. For example, in just 12 years between 1974 and 1986 there was a two to fourfold increase in diverticular disease incidence in Japan [4]. In addition, an autopsy study reported a prevalence of 1% among Japanese in Japan, but a prevalence of 50% among Japanese in the US [5], and other migration studies have suggested an increased risk with a longer duration since settlement [6]. In the US, 65% of adults will develop diverticulosis by age 80 years [7, 8]. These observations suggest that modifiable risk factors are of major importance for the development of diverticular disease.

A diet low in fiber and high in red meat has been associated with increased risk of diverticular disease [9–11]. Overweight and obesity have been associated with increased risk of diverticular disease as well [12]. Several case–control [13–15] and cohort studies [11, 16–21], but not all [22] have reported increased risk of diverticular disease with greater body mass index. However, studies have differed with regard to the strength of the reported associations with risk estimates ranging between 33 and 340% increases in the relative risk among obese persons [11, 16–20]. On the other hand, some studies have suggested an inverse association between physical activity and diverticular disease [18, 22–24], however, not all studies found a significant association [11, 16, 21]. Because relatively few risk factors for diverticular disease have been firmly established and because clarification of potential preventive measures that could modify the risk of the disease is important, we conducted a systematic review and meta-analysis of prospective studies of body mass index, physical activity, and the risk of diverticular disease. Our specific aims were to examine the strength of the association and the shape of the dose–response relationship between increasing adiposity and physical activity and diverticular disease risk.

## Methods

### Search strategy

We searched PubMed and Embase databases for relevant studies up to February 19, 2016 using the following search terms as part of a larger project on diverticular disease risk factors (“body mass index” OR BMI OR overweight OR obesity OR anthropometry OR fatness OR “body fatness” OR “abdominal fatness” OR “abdominal obesity” OR “waist circumference” OR “waist-to-hip ratio” OR “waist-to-height ratio” OR “hip circumference” OR adiposity OR weight OR “weight gain” OR “weight change” OR “weight loss” OR “body size” OR “physical activity” OR exercise OR sports OR walking OR biking OR running OR fitness OR “exercise test” OR inactivity OR sedentary OR fiber OR fibre OR diet OR meat OR “red meat” OR “processed meat” OR beef OR pork OR lamb OR smoking OR tobacco OR risk factor OR risk factors) AND (“diverticular disease” OR diverticulitis OR “diverticular bleeding” OR diverticula OR diverticulosis) AND (“case-control” OR retrospective OR cohort OR cohorts OR prospective OR longitudinal OR “follow-up” OR “cross-sectional” OR trial OR “odds ratio” OR “relative risk” OR “hazard ratio” OR “incidence rate ratio” OR “risk ratio”). In addition, we searched the reference lists of all studies included in the analysis. We followed the PRISMA criteria for reporting of systematic reviews and meta-analyses [25].

### Study selection

Prospective studies of the association between BMI or other adiposity measures and physical activity and diverticular disease risk were included. Adjusted relative risk estimates (hazard ratio, risk ratio, odds ratio) with their 95% confidence intervals had to be available in the publication, and for the dose–response analysis, the exposure had to be quantified for at least three categories and the total number of cases and person-years had to be available. We identified nine relevant full-text publications. A list of the excluded studies and the exclusion reasons is provided in Supplementary Table 1.

### Data extraction

We extracted from each study the following: the first author’s last name, publication year, country where the study was conducted, study name, follow-up period, sample size, gender, age, number of cases, assessment method of anthropometric factors (measured vs. self-reported), assessment method of the outcome, adiposity measure, physical activity type, RRs and 95% CIs, and variables adjusted for in the analysis. Data were extracted by one reviewer (DA) and checked for accuracy by a second reviewer (AS). Any disagreements were resolved by discussion.

### Statistical analysis

Summary RRs and 95% CIs for a 5 unit increment in BMI and for a high vs. low physical activity level were estimated using a random effects model [26]. The average of the natural logarithm of the RRs was estimated and the RR from each study was weighted using random effects weights. A two-tailed *p* < 0.05 was considered statistically significant.

We used the method described by Greenland and Longnecker [27] for the dose–response analysis and study-specific slopes (linear trends), and 95% CIs were computed from the natural logs of the RRs and CIs across categories of BMI. The mean or median BMI level in each category was assigned to the corresponding relative risk for each study, and for studies that reported these measures by ranges we estimated the midpoint for each category by calculating the average of the upper and lower cut-off points for each category. When extreme categories were open-ended, we used the width of the adjacent interval to calculate an upper or a lower cut-off point. We examined a potential nonlinear dose–response relationship between BMI and diverticular disease using fractional polynomial models [28]. We determined the best fitting second-order fractional polynomial regression model, defined as the one with the lowest deviance. A likelihood ratio test was used to assess the difference between the nonlinear and linear models to test for nonlinearity [28]. Study quality was assessed using the Newcastle-Ottawa scale which rates studies according to selection, comparability, and outcome assessment with a score from 0 to 9 [29].

Subgroup and meta-regression analyses were conducted to investigate potential sources of heterogeneity and heterogeneity between studies was quantitatively assessed by the *Q* test and *I* ^2^ [30]. Small-study effects, such as publication bias, were assessed by inspecting the funnel plots for asymmetry and with Egger’s test [31] and Begg’s test [32], with the results considered to indicate small-study effects when *p* < 0.10. Sensitivity analyses excluding one study at a time were conducted to clarify whether the results were robust to the influence of individual studies.

## Results

**Fig. 1:**
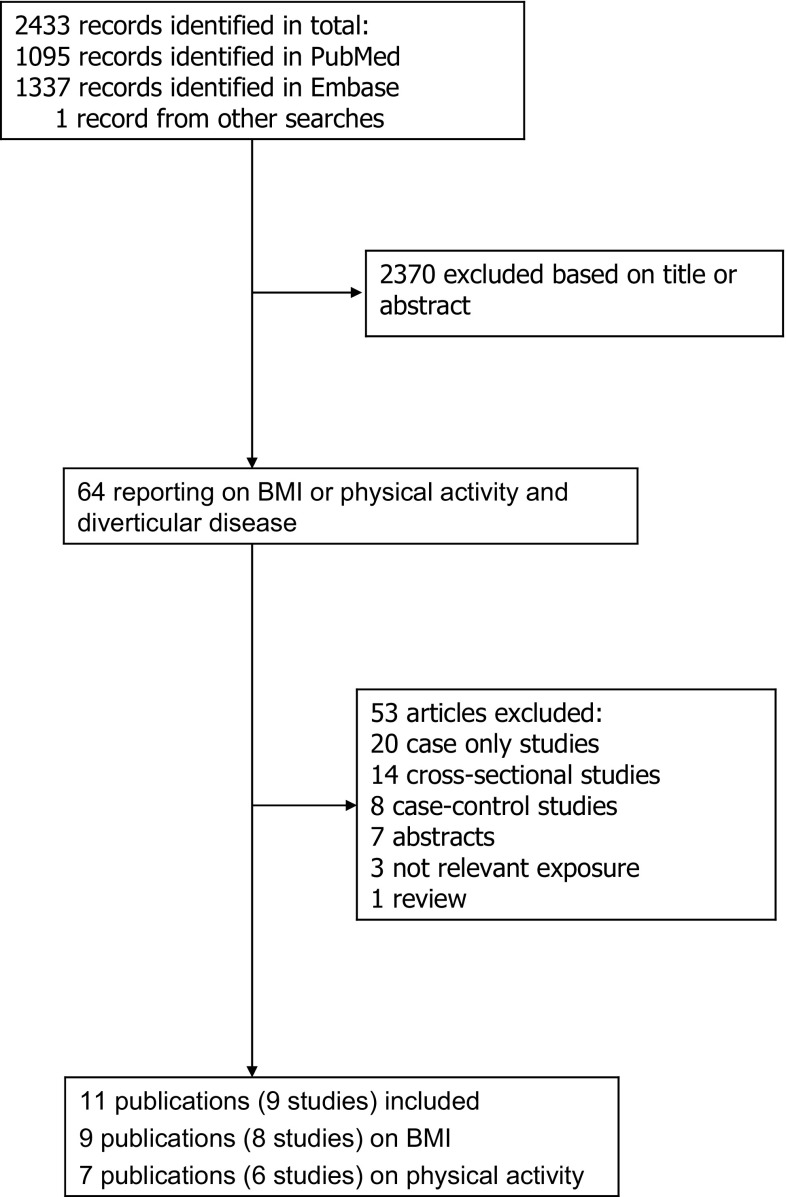
Flow-chart of study selection of BMI and physical activity in relation to diverticular disease

Out of 2433 records identified by the search, we included eleven publications (nine studies) in total [11, 14, 16–24], six prospective studies [11, 16, 18–20, 22] in the analyses of BMI and diverticular disease incidence, two studies in the analysis of BMI and diverticulitis [17, 21], and three studies [14, 17, 18] on BMI and diverticular disease complications and five studies [11, 16, 18, 22, 24] on physical activity and diverticular disease incidence and two studies on vigorous physical activity and diverticulitis [21, 23] (Tables 1, 2; Fig. 1). Characteristics of the included studies are provided in Tables 1 and 2. Of the studies on BMI, five were from Europe, one from the US, and one from Australia, and of the studies on physical activity, four were from Europe and two were from the US (Table 1).

**Table 1:** Prospective studies of body mass index and diverticular disease incidence

References, publication year, country/region	Study name	Follow-up period	Study size, gender, age, number of cases	Assessment of weight and height	Study quality	Exposure	Description of quantiles of categories	RR (95% CI)	Adjustment for confounders
Aldoori WH et al. [22], 1995, USA	Health Professionals Follow-up Study	1988–1992, 4 years of follow-up	47,678 men, age 42–77 years: 382 diverticular disease cases	Self-reported (validated)	7	BMI	22.0	1.00	Age, physical activity, dietary fiber, total fat
							23.7	1.15 (0.81–1.62)	
							25.1	1.23 (0.87–1.72)	
							26.5	1.23 (0.88–1.72)	
							29.4	1.22 (0.87–1.70)	
Rosemar A et al. [16], 2008, Sweden	The Multifactorial Prevention Trial	1970–1973–1998, max 28 years of follow-up	7494 men, age 47–55 years: 112 hospitalizations for diverticular disease	Measured	8	BMI	<20	3.0 (0.7–12.5)	Age, smoking, diastolic blood pressure
							20–22.5	1.0	
							22.5–25	2.3 (0.9–6.0)	
							25–27.5	3.0 (1.2–7.6)	
							27.5–30	3.2 (1.2–8.6)	
							>30	4.4 (1.6–12.3)	
Strate LL et al. [17], 2009, USA	Health Professionals Follow-up Study	1986–2004, ~15.5 years of follow-up	47,228 men, age 40–75 years: 801 diverticulitis cases 383 cases of diverticular bleeding	Self-reported	7	BMI, diverticulitis	<21	1.00	Age, study period, calories, fat, fiber, red meat, physical activity, NSAID use, acetaminophen
							21–22.9	1.29 (0.77–2.14)	
							23–24.9	1.40 (0.86–2.28)	
							25–27.4	1.48 (0.92–2.39)	
							27.5–29.9	1.58 (0.97–2.59)	
							≥30.0	1.78 (1.08–2.94)	
						BMI, diverticular bleeding	<21	1.00	
							21–22.9	1.68 (0.75–3.76)	
							23–24.9	1.83 (0.85–3.97)	
							25–27.4	2.38 (1.11–5.09)	
							27.5–29.9	1.91 (0.87–4.23)	
							≥30.0	3.19 (1.45–7.00)	
						Waist circumference, diverticulitis	≤34.25 inches	1.00	
							34.5–36	1.22 (0.92–1.62)	
							36.25–37.75	1.22 (0.91–1.64)	
							38–40	1.20 (0.91–1.59)	
							≥40.25	1.56 (1.18–2.07)	
						Waist circumference, diverticular bleeding	≤34.25 inches	1.00	
							34.5–36	0.91 (0.57–1.44)	
							36.25–37.75	1.44 (0.93–2.23)	
							38–40	1.36 (0.90–2.08)	
							≥40.25	1.96 (1.30–2.97)	
						WHR, diverticulitis	<0.89	1.00	
							0.89–0.92	1.22 (0.92–1.63)	
							0.93–0.95	1.30 (0.97–1.73)	
							0.96–0.98	1.41 (1.06–1.87)	
							>0.98	1.62 (1.23–2.14)	
						WHR, diverticular bleeding	<0.89	1.00	
							0.89–0.92	1.05 (0.66–1.67)	
							0.93–0.95	1.37 (0.88–2.13)	
							0.96–0.98	1.43 (0.92–2.20)	
							>0.98	1.91 (1.26–2.90)	
Crowe FL et al. [11], 2011, United Kingdom	EPIC-Oxford	1993–1999–2009, 11.6 years of follow-up	47,033 men and women, age ≥ 20 years: 812 diverticular disease cases	Measured and self-reported	7	BMI	<20	0.63 (0.42–0.93)	Age, sex, method of recruitment, region of residence, smoking
							20.0–22.5	1.00	
							22.5–25.0	1.23 (1.00-1.51)	
							25.0–27.5	1.54 (1.24–1.92)	
							≥27.5	1.67 (1.34–2.08)	
Humes DJ et al. [14], 2011, United Kingdom	UK General Practice Research Database	1990–2005, ~15 years of follow-up	899 perforated diverticular disease cases 8980 population controls Age ≥ 45 years	Measured/ self-reported	7	BMI	<25	1.00	Age, sex
							25–29.9	0.98 (0.82–1.17)	
							≥30	1.34 (1.06–1.69)	
Hjern F et al. [18], 2012, Sweden	Swedish Mammography Cohort	1997–2009, 12 years of follow-up	36,592 women, mean age 61.8 years: 626 diverticular disease cases	Self-reported	9	BMI, diverticular disease requiring hospitalization	<20	1.01 (0.70–1.45)	Age, dietary fiber, diabetes, hypertension, acetylsalicylic acid, NSAIDs, steroid medication, alcohol, smoking status, physical activity, education
							20–24.99	1.00	
							25–29.99	1.29 (1.08–1.54)	
							≥30	1.33 (1.03–1.72)	
						BMI, diverticular disease with abscess/perforation	<20	2.24 (1.10–4.56)	
							20-24.99	1.00	
							25-29.99	1.49 (0.94–2.39)	
							≥30	2.00 (1.08–3.73)	
Korda RJ et al. [19], 2012, Australia	The 45 and Up Study	2006–2009, 2.3 years of follow-up	246,361 men and women, age ≥ 45 years: 804 diverticular disease hospitalizations	Self-reported	5	BMI, age 45–64 years	18.5–<25.0	1.00	Age, sex, region of residence, household income, smoking, alcohol, private health insurance status
							25.0–<30.0	1.42 (1.06–1.90)	
							≥30.0	2.18 (1.63–2.91)	
						BMI, age 65–79 years	18.5–<25.0	1.00	
							25.0–<30.0	1.20 (0.91–1.58)	
							≥30.0	1.97 (1.47–2.63)	
						BMI, age ≥ 80 years	18.5–<25.0	1.00	
							25.0–<30.0	1.40 (1.01–1.95)	
							≥30.0	1.57 (1.00-2.48)	
Reeves GK et al. [20], 2014, United Kingdom	Million Women’s Study	1996–2001–2008, 9.2 years of follow-up	1,251,619 women, age 50–64 years: 26,179 diverticular disease hospitalizations	Self-reported	7	BMI	<22.5	0.89 (0.86–0.91)	Age, geographical region, SES, age at 1st birth, parity, smoking status, alcohol intake, physical activity, time since menopause, HRT use
							22.5–24.9	1.00 (0.98–1.03)	
							25.0-29.9	1.23 (1.21–1.26)	
							30.0-34.9	1.43 (1.38–1.47)	
							≥35.0	1.56 (1.49–1.63)	
							Per 5 units	1.22 (1.20–1.24)	
Jamal Talabani A et al. [21], 2016, Norway	The North-Trondelag Health Study	1995–1997–1998–2012, ~14 years of follow-up	42,570 men and women, age ≥20 years: 358 acute diverticulitis cases	Measured	8	BMI, women	<25.0	1.00	Age, hard physical activity, smoking status, problems with breathlessness, problems with constipation, type of bread, education, living area
							25.0-29.9	1.25 (0.90–1.73)	
							≥30.0	2.06 (1.46–2.91)	
						BMI, men	<25.0	1.00	
							25.0-29.9	1.46 (0.92–2.32)	
							≥30.0	2.58 (1.53–4.34)	

HRT = hormone replacement therapy, NSAID = nonsteroidal anti-inflammatory drugs, SES = socioeconomic status

**Table 2:** Studies of physical activity and diverticular disease

References, publication year, country	Study name	Study period	Number of participants, gender, age, number of cases	Study quality	Physical activity exposure	Quantity	Relative risk (95% confidence interval)	Adjustment for confounders
Aldoori WH et al. [22], 1995, USA	Health Professionals Follow-up Study	1988–1992, 4 years of follow-up	47,678 men, age 42–77 years: 382 diverticular disease cases	7	Total leisure-time physical activity	0.9 MET-h/week	1.00	Age, dietary fiber, total fat
						4.8	0.91 (0.68–1.21)	
						11.3	0.71 (0.52–0.97)	
						22.6	0.74 (0.54–1.01)	
						46.8	0.63 (0.45–0.88)	
					Nonvigorous activity	0.1 MET-h/week	1.00	
						1.4	1.15 (0.84–1.58)	
						3.4	0.79 (0.56–1.12)	
						7.6	1.09 (0.79–1.49)	
						20.8	0.93 (0.67–1.69)	
					Vigorous activity	0 MET-h/week	1.00	
						3.5	0.78 (0.60–1.02)	
						15.0	0.88 (0.67–1.15)	
						41.0	0.60 (0.41–0.87)	
Rosemar A et al. [16], 2008, Sweden	The Multifactor Primary Prevention Trial	1970–1973–1998, 28 years of follow-up	7494 men, age 47–55 years: 112 diverticular disease hospitalizations	8	Leisure-time physical activity	Sedentary	1.0	Age, smoking, diastolic blood pressure
						Moderate	0.9 (0.6–1.4)	
						Active	1.2 (0.7–2.0)	
Williams PT et al. [24], 2009, USA	National Runners’ Health Study	1991–1994–1999–2002, 7.7 years of follow-up	9072 men and 1664 women, age ≥ 50 years: 127/21 diverticular disease cases	5	Running distance	Per 1 km/day	0.938, *p* = 0.04	Age, sex, pack-years of cigarette smoking, meat, fish, fruit, alcohol
						≥8 vs. <2 km/day	0.52, *p* = 0.05	
						Per 1 km/day	0.945, *p* = 0.08	+ BMI
Strate LL et al. [12], 2009, USA	Health Professionals Follow-up Study	1986–2004, 18 years of follow-up	47,228 men, age 40–75 years: 800 diverticulitis cases 383 diverticular bleeding cases	7	Total leisure-time physical activity, diverticulitis	≤8.2 METs/week	1.00	Age, study period, sedentary behavior, BMI, NSAID use, acetaminophen, dietary fat, fiber, red meat, combined nut/corn/popcorn consumption, total energy
						8.3–19.0	1.08 (0.87–1.34)	
						19.1–33.5	0.97 (0.77–1.21)	
						33.6–57.3	0.98 (0.78–1.23)	
						≥57.4	0.75 (0.58–0.95)	
					Vigorous physical activity	0 METs/week	1.00	
						0.1–4.0	0.91 (0.74–1.12)	
						4.1–10.0	0.99 (0.81–1.22)	
						10.1–28.0	0.89 (0.72–1.10)	
						≥28.0	0.66 (0.51–0.86)	
					Nonvigorous physical activity	0–2.9 METs/week	1.00	
						3.0–7.9	0.94 (0.75–1.18)	
						8.0–15.9	0.91 (0.73–1.15)	
						16.0–29.9	0.90 (0.71–1.13)	
						≥30.0	0.96 (0.76–1.21)	
					Total leisure-time physical activity, diverticular bleeding	≤8.2 METs/week	1.00	
						8.3–19.0	0.91 (0.68–1.22)	
						19.1–33.5	0.71 (0.52–0.98)	
						33.6–57.3	0.66 (0.48–0.92)	
						≥57.4	0.54 (0.38–0.77)	
					Vigorous physical activity	0 METs/week	1.00	
						0.1–4.0	1.04 (0.79–1.37)	
						4.1–10.0	0.70 (0.51–0.97)	
						10.1–28.0	0.81 (0.59–1.10)	
						≥28.0	0.61 (0.41–0.90)	
					Nonvigorous physical activity	0–2.9 METs/week	1.00	
						3.0–7.9	0.90 (0.65–1.25)	
						8.0–15.9	0.89 (0.64–1.23)	
						16.0–29.9	0.96 (0.70–1.33)	
						≥30.0	0.77 (0.55–1.07)	
Crowe FL et al. [11], 2011, United Kingdom	EPIC-Oxford Study	1993–1999–2009, 11.6 years of follow-up	47,033 men and women, age ≥ 20 years: 812 diverticular disease cases	7	Physical activity	Inactive	1.00	Age, sex, method of recruitment, region of residence, smoking
						Active	0.87 (0.74–1.02)	
Hjern F et al. [18], 2012, Sweden	Swedish Mammography Cohort	1997–2009, 12 years of follow-up	36,592 women, mean age 61.8 years: 626 diverticular disease cases	9	Physical activity, hospital requiring diverticular disease Physical activity, diverticular disease with abscess/perforation	≤ 30 min/d	1.42 (1.18–1.69)	Age, dietary fiber, diabetes mellitus, hypertension, acetylsalicylic acid, NSAID use, steroid medication, alcohol, smoking status, BMI, education
						> 30 min/d	1.00	
						≤ 30 min/d	0.77 (0.47–1.27)	
						> 30 min/d	1.00	
Jamal Talabani A et al. [21], 2016, Norway	The North-Trondelag Health Study	1995–1997–1998–2012, ~14 years of follow-up	42,570 men and women, age ≥ 20 years: 358 acute diverticulitis cases	7	Hard physical activity, women Hard physical activity, men	<1 h/week	1.00	Age, BMI, smoking status, problems with breathlessness, problems with constipation, type of bread, education, living area
						≥1	0.67 (0.39–1.15)	
						<1 h/week	1.00	
						≥1	1.03 (0.67–1.57)	

*NSAID* nonsteroidal anti-inflammatory drugs

### BMI

**Fig. 2:**
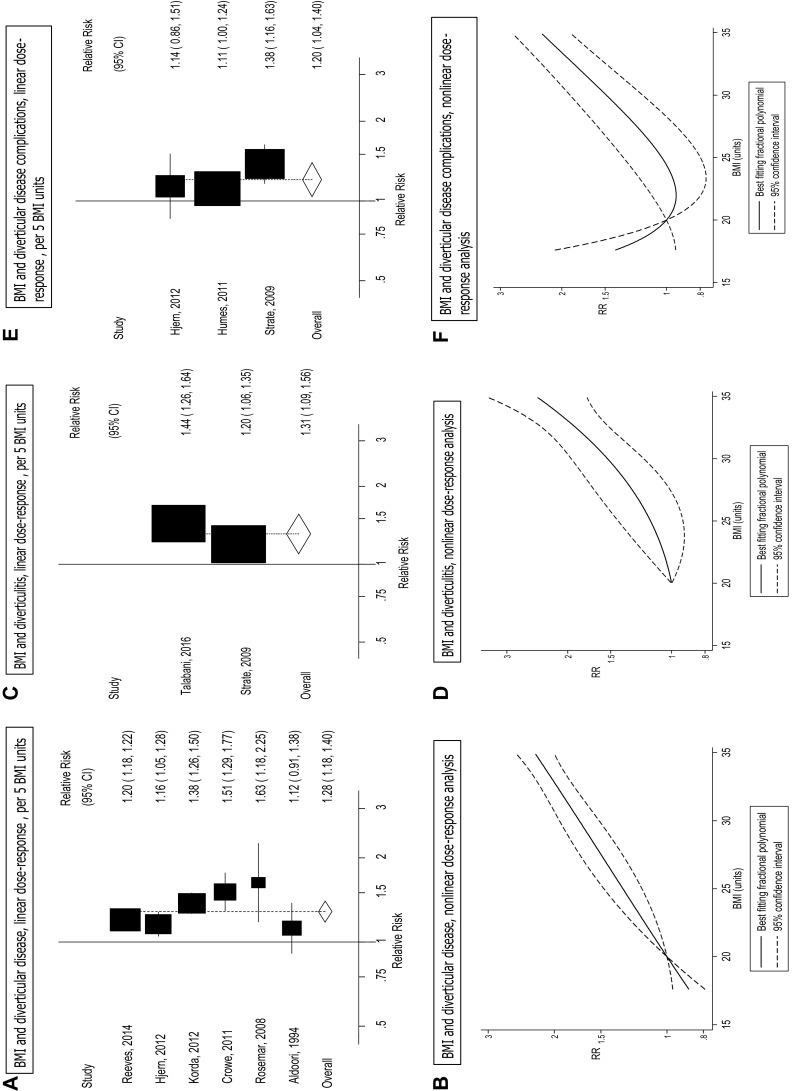
BMI and diverticular disease, diverticulitis, and diverticular disease complications, linear dose–response analyses (per 5 BMI units), and nonlinear dose–response analyses

Six studies [11, 16, 18–20, 22] were included in the analysis of BMI and diverticular disease risk and included 28,915 cases and 1,636,777 participants. The summary RR for the highest vs. the lowest BMI was 1.78 (95% CI: 1.48–2.14, *I* ^2^ = 66.0%, *p*_heterogeneity_ = 0.01). In the dose–response analysis, the summary RR for a 5 unit increase in BMI was 1.28 (95% CI: 1.18–1.40, *I* ^2^ = 77%, *p*_heterogeneity_=0.001) (Fig. 2a). There was no evidence of small-study bias (Egger’s test, *p* = 0.21 or with Begg’s test, *p* = 0.71). There was no evidence of nonlinearity or threshold levels between BMI and diverticular disease (*p*_nonlinearity_ = 0.22), and the risk increased monotonically with increasing BMI even within the normal BMI range (Fig. 2b, Supplementary Table 2).

Two studies were included in the analysis of BMI and diverticulitis [17, 21] and included 1159 cases and 89,798 participants. The summary RR for the highest vs. lowest BMI category was 2.09 (95% CI: 1.63–2.68, *I* ^2^ = 0%, *p*_heterogeneity_ = 0.47). In the dose–response analysis, the summary RR for a 5 unit increase in BMI was 1.31 (95% CI: 1.09–1.56, *I* ^2^ = 74%, *p*_heterogeneity_ = 0.05) (Fig. 2c). There was no evidence of nonlinearity or threshold levels between BMI and diverticulitis (*p*_nonlinearity_ = 0.25) (Fig. 2d, Supplementary Table 2).

Three studies [14, 17, 18] were included in the analysis of BMI and risk of diverticular disease complications (bleeding, perforation, or abscess) and included 2326 cases and 93,699 participants. The summary RR for a 5 unit increase in BMI was 1.20 (95% CI: 1.04–1.40, *I* ^2^ = 56%, *p*_heterogeneity_ = 0.10) (Fig. 2e). There was evidence of a nonlinear association between BMI and diverticular disease complications (*p*_nonlinearity_ < 0.0001), with the lowest risk being observed at a BMI of 22 (Fig. 2f, Supplementary Table 2). Because only one study [17] reported data regarding waist circumference, waist-to-hip ratio, and diverticular disease incidence, we were not able to conduct a meta-analysis of these measures.

### Physical activity

**Fig. 3:**
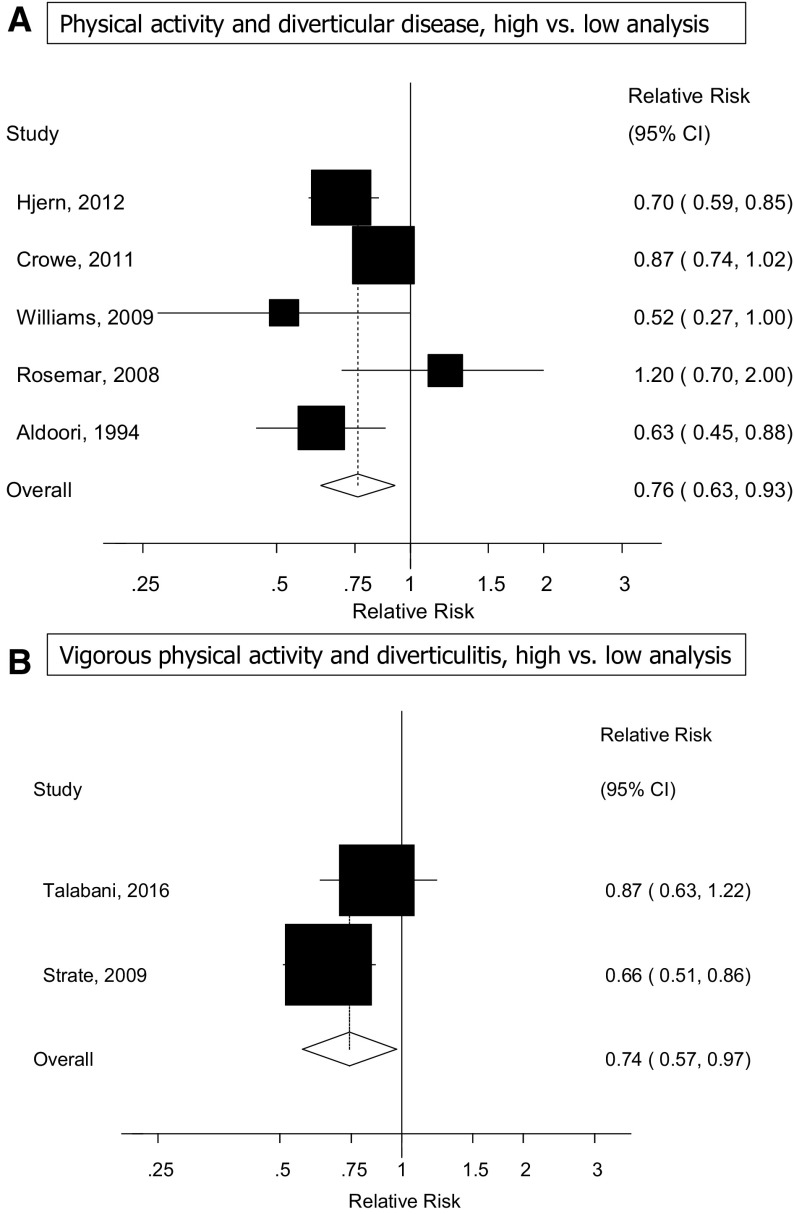
High vs. low analysis of physical activity and diverticular disease and of vigorous physical activity and diverticulitis

Five cohort studies [11, 16, 18, 22, 24] were included in the analysis of physical activity and diverticular disease risk and included 2080 cases and 147,869 participants. The summary RR for high vs. low activity was 0.76 (95% CI: 0.63–0.93, *I* ^2^ = 54%, *p*_heterogeneity_ = 0.07) (Fig. 3a). The heterogeneity was slightly reduced when excluding one study [11], but the overall estimate was similar (summary RR = 0.72, 95% CI: 0.56–0.91, *I* ^2^ = 42%, *p*_heterogeneity_ = 0.16). Because of differences in the way the studies reported the data, it was not possible to conduct a dose–response analysis for physical activity.

Two cohort studies [21, 23] reported on vigorous physical activity and diverticulitis and included 1158 cases and 89,798 participants. The summary RR for high vs. low vigorous physical activity was 0.74 (95% CI: 0.57–0.97, *I* ^2^ = 39.5%, *p*_heterogeneity_ = 0.20) (Fig. 3b).

### Subgroup analyses, sensitivity analyses, and study quality

The association between BMI and diverticular disease persisted in most, but not all subgroup analyses defined by sex, duration of follow-up, assessment of weight and height, outcome assessment, geographic location, study quality, and adjustment for confounding factors, and there was little evidence of heterogeneity between any of these subgroups (Table 3). Only in the subgroup analysis stratified by adjustment for physical activity was there evidence of heterogeneity, with a weaker, but still statistically significant association among studies with adjustment for physical activity compared to studies without such adjustment. The association between physical activity and diverticular disease also persisted in most subgroup analyses, and there was no evidence of heterogeneity between any of these subgroups (Table 4). Mean (median) study quality scores were 7.2 (7.0) for the studies on BMI and diverticular disease and 7.2 (7.0) for the studies on physical activity and diverticular disease.

**Table 3:** Subgroup analyses of BMI and diverticular disease

	BMI				
	*n*	RR (95% CI)	*I* ^2^ (%)	*P*_h_^a^	*P*_h_^b^
All studies	6	1.28 (1.18–1.40)	77.0	0.001	
Sex					
Men	2	1.14 (0.94–1.39)	0	0.53	0.02/NC^c^
Women	2	1.20 (1.18–1.22)	0	0.48	
Men and women	2	1.41 (1.30–1.52)	4.9	0.31	
Assessment of weight/height					
Measured	1	1.63 (1.18–2.25)			0.80
Self-reported	4	1.23 (1.14–1.32)	70.9	0.02	
Measured and self-reported	1	1.51 (1.29–1.77)			
Assessment of the diverticular disease					
Linkage to medical records/hospitalizations	5	1.31 (1.19–1.44)	81.1	<0.0001	0.39
Self-report (validated)	1	1.12 (0.91–1.38)			
Duration of follow-up					
<10 years of follow-up	3	1.25 (1.12–1.39)	79.4	0.008	0.48
≥10 years of follow-up	3	1.38 (1.10–1.73)	80.7	0.006	
Geographic location					
Europe	4	1.28 (1.15–1.43)	75.4	0.007	0.48
America	1	1.12 (0.91–1.38)			
Asia	1	1.38 (1.26–1.50)			
Number of cases					
Cases < 250	1	1.40 (0.73–2.67)			0.64
Cases 250 < 500	1	1.12 (0.91–1.38)			
Cases ≥ 500	4	1.28 (1.17–1.41)	83.2	<0.0001	
Study quality					
0–3 points	0				0.61
4–6	1	1.38 (1.26–1.50)			
7–9	5	1.26 (1.14–1.38)	68.4	0.01	
Adjustment for confounders					
Age					
Yes	6	1.28 (1.18–1.40)	77.0	0.001	NC
No	0				
Education					
Yes	1	1.16 (1.05–1.28)			0.39
No	5	1.32 (1.18–1.48)	81.0	<0.0001	
Alcohol					
Yes	3	1.24 (1.14–1.35)	79.7	0.007	0.40
No	3	1.39 (1.11–1.73)	67.8	0.05	
Smoking					
Yes	5	1.31 (1.19–1.44)	81.1	<0.0001	0.39
No	1	1.20 (1.06–1.35)			
Diabetes					
Yes	1	1.16 (1.05–1.28)			0.39
No	5	1.32 (1.18–1.48)	81.0	<0.0001	
Aspirin use					
Yes	1	1.16 (1.05–1.28)			0.39
No	5	1.32 (1.18–1.48)	81.0	<0.0001	
NSAID use					
Yes	1	1.16 (1.05–1.28)			0.39
No	5	1.32 (1.18–1.48)	81.0	<0.0001	
Acetaminophen					
Yes	0				NC
No	6	1.28 (1.18–1.40)	77.0	0.001	
Physical activity					
Yes	3	1.20 (1.18–1.22)	0	0.63	0.01
No	3	1.42 (1.32–1.53)	0	0.40	
Meat					
Yes	0				NC
No	6	1.28 (1.18–1.40)	77.0	0.001	
Fiber					
Yes	2	1.15 (1.05–1.26)	0	0.78	0.17
No	4	1.37 (1.20–1.56)	85.4	<0.0001	

*N* denotes the number of risk estimates, *NC* not calculable

^a^ *P* for heterogeneity within each subgroup

^b^ *P* for heterogeneity between subgroups

^c^ *P* for heterogeneity between men and women (excluding studies with both genders combined)

**Table 4:** Subgroup analyses of physical activity and diverticular disease

		Physical activity				
		*n*	RR (95% CI)	*I* ^*2*^ (%)	*P*_h_^a^	*P*_h_^b^
All studies		5	0.76 (0.63–0.93)	54.3	0.07	
Sex						
Men		2	0.84 (0.45–1.58)	75.7	0.04	0.86/0.78^c^
Women		1	0.70 (0.59–0.85)			
Men and women		2	0.74 (0.47–1.18)	55.3	0.14	
Duration of follow-up						
<10 years of follow-up		2	0.61 (0.45–0.82)	0	0.61	0.35
≥10 years of follow-up		3	0.83 (0.66–1.04)	62.8	0.07	
Geographic location						
Europe		3	0.83 (0.66–1.04)	62.8	0.07	0.46
America		2	0.61 (0.45–0.82)	0	0.61	
Asia		0				
Number of cases						
Cases < 250		2	0.81 (0.36–1.83)	73.8	0.05	0.76
Cases 250 < 500		1	0.63 (0.45–0.88)			
Cases ≥ 500		2	0.78 (0.63–0.97)	67.5	0.08	
Study quality						
0–3 points						
4–6		1	0.52 (0.27–1.00)			
7–9		4	0.79 (0.64–0.96)	58.6	0.06	
Adjustment for confounders						
Age						
Yes		5	0.76 (0.63–0.93)	54.3	0.07	NC
No		0				
Education						
Yes		2	0.78 (0.63–0.97)	67.5	0.08	0.98
No		3	0.74 (0.47–1.16)	61.5	0.07	
Alcohol						
Yes		2	0.69 (0.57–0.82)	0	0.39	0.17
No		3	0.84 (0.63–1.11)	58.2	0.09	
Smoking						
Yes		4	0.80 (0.64–0.99)	57.5	0.07	0.83
No		1	0.63 (0.45–0.88)			
Diabetes						
Yes		1	0.70 (0.59–0.85)			0.32
No		4	0.79 (0.60–1.04)	55.7	0.08	
Aspirin use						
Yes		1	0.70 (0.59–0.85)			0.32
No		4	0.79 (0.60–1.04)	55.7	0.08	
NSAID use						
Yes		1	0.70 (0.59–0.85)			0.22
No		4	0.79 (0.60–1.04)	55.7	0.08	
Acetaminophen						
Yes		0				NC
No		5	0.76 (0.63–0.93)	54.3	0.07	
BMI						
Yes		1	0.70 (0.59–0.85)			0.22
No		4	0.79 (0.60–1.04)	55.7	0.08	
Meat						
Yes		1	0.52 (0.27–1.00)			0.46
No		4	0.79 (0.64–0.96)	58.6	0.06	
Fiber						
Yes		2	0.68 (0.58–0.80)	0	0.59	0.22
No		3	0.86 (0.61–1.19)	47.6	0.15	

*N* denotes the number of risk estimates, *NC* not calculable

^a^ *P* for heterogeneity within each subgroup

^b^ *P* for heterogeneity between subgroups

^c^ *P* for heterogeneity between men and women (excluding studies with both genders combined)

## Discussion

To our knowledge, this is the first meta-analysis of BMI and physical activity in relation to risk of diverticular disease. There was a 28% increase in the relative risk of diverticular disease, a 31% increase in the relative risk of diverticulitis, and a 20% increase in the relative risk of diverticular disease complications for each 5 unit increase in BMI. In addition, there was a 24% reduction in risk of diverticular disease incidence for the highest vs. lowest level of physical activity and a 26% reduction in the risk of diverticulitis for the highest vs. lowest level of vigorous physical activity. The association between BMI and diverticular disease incidence appeared to be linear with a 15% increase in risk with a BMI of 22.5 kg/m^2^ compared to 20 kg/m^2^, a 50% increase in risk among overweight subjects, and an approximately 2- to 3-fold increase in risk in obese and severely obese subjects, respectively, while for diverticular disease complications there was an indication of nonlinearity and the lowest risk was observed at a BMI of 22 kg/m^2^, and a slight increase in risk was observed in the underweight BMI category. It is unclear whether the increased risk at low BMI represents reverse causation or simply is a chance finding as the number of studies in that analysis was small and because there was only one study which suggested an increased risk with a low BMI.

Our meta-analysis has some limitations, which may affect the interpretation of the results. It is possible that the positive association between BMI and diverticular disease could be due to unmeasured or residual confounding by other lifestyle factors, such as higher intakes of red meat, higher prevalence of smoking, or lower intake of dietary fiber. The results for BMI and physical activity persisted when stratified by adjustment for dietary fiber, meat, and smoking, and also persisted when mutually adjusted. Although there was evidence of heterogeneity in the analyses of BMI and physical activity in relation to diverticular disease risk, for BMI the heterogeneity appeared to be due to differences in the magnitude of the association rather than to differences in the presence or absence of an association, as all studies found increased risk, while for physical activity there was a moderate heterogeneity, which was partly explained by one outlying study. Most studies relied on self-reported height and weight, and although there may be some under-reporting of weight and over-reporting of height, most studies have found a high correlation between self-reported and measured height and weight [33]. Differences between studies in the identification and diagnosis of diverticular disease cases may also be a limitation as some cases may be asymptomatic or may only have mild symptoms. It is possible that detection bias could have influenced the findings because individuals with a greater BMI are generally more likely to be admitted to hospital and might also be more likely to undergo examinations that could lead to the diagnosis of diverticular disease. Most of the diverticular disease cases in the studies included in this meta-analysis would most likely have been symptomatic because the studies identified cases through record linkages to databases on hospitalization or death from diverticular disease [11, 16, 18–20], and one study identified cases by self-report of symptomatic diverticular disease [22]. In addition, we found similar associations between BMI and diverticular disease as with diverticulitis and diverticular disease complications. Although we cannot exclude the possibility that the association between BMI and asymptomatic diverticular disease might differ from the current findings, establishing the association with more “advanced” disease might be more relevant in terms of preventing severe complications from diverticular disease. Two studies which conducted validation studies of the diagnosis found that 95–96% of cases were correctly identified by self-report or linkage to patient registers [9, 18], and we found no heterogeneity when studies were stratified by the assessment of the outcome. Because of the prospective design of the included studies, any misclassification of the outcome would likely lead to an underestimation of the association between BMI and physical activity and diverticular disease risk. Although meta-analyses of published literature may be susceptible to small-study effects, we found no evidence of small-study effects with either Egger’s test or Begg’s test or when visually inspecting the funnel plots; however, the modest number of available studies is a limitation.

The studies on physical activity and diverticular disease reported the data using different underlying measures (MET-hours per week, or in <3 categories or without quantifying the physical activity level in each category), and for this reason we were not able to conduct dose–response analyses of physical activity. This appears to be a common problem in studies on physical activity and health outcomes [34–37] and emphasizes the need for a more thorough and standardized approach to analyses and reporting of data on physical activity and different health outcomes. Further studies are therefore needed to characterize the dose–response relationship between physical activity and specific subtypes and intensities of physical activity in relation to diverticular disease, preferably using an underlying metric that could be combined with other published studies, for example using MET-hours per week and/or hours per week of activity.

Our meta-analysis also has several strengths, including increased statistical power due to a large sample size, the detailed search strategy, comprehensive analyses including both linear and nonlinear dose–response analyses for BMI, and several sensitivity analyses.

Little is known about the biological mechanisms that could explain an association between adiposity and diverticular disease risk. Adipose tissue secretes cytokines that may contribute to diverticular inflammation. The bacterial flora of obese and lean subjects may differ [38], and some evidence suggests that the bacterial flora may be important for the development of diverticular disease [39]. Physical activity could reduce the risk of diverticular disease by preventing overweight and obesity, by maintaining gastrointestinal motor function, decreasing intra-colonic pressure, reducing the transit time, and through neuroendocrine changes [40]. Nevertheless, further research is clearly needed to firmly establish the underlying biological mechanisms.

In conclusion, the current meta-analysis suggests that excess weight and low physical activity are risk factors for diverticular disease. The current findings have important public health implications as they add diverticular disease to the list of conditions that appear to be associated with adiposity and low physical activity. The findings support recommendations for overall health to avoid excess weight and to be physically active. Further studies are needed to assess the association between different measures of adiposity as well as subtypes and intensities of physical activity in relation to diverticular disease, diverticulitis, and the associated complications, and any further studies should report data on physical activity using a measure (MET-hours/week or hours/week) that can be combined with other published studies for future dose–response analyses.

## Electronic supplementary material

Below is the link to the electronic supplementary material.

Supplementary material 1 (DOC 59 KB)

Supplementary material 2 (DOCX 52 KB)
