# Supplementary material for: Body mass index and physical activity and the risk of diverticular disease: a systematic review and meta-analysis of prospective studies
Source: Eur J Nutr. 2017 Apr 9;56(8):2423–38. doi: 10.1007/s00394-017-1443-x (PMC5682875; doi:10.1007/s00394-017-1443-x)
Supplement: Supplementary file 2 — Supplementary material 2 (DOCX 52 KB) [file 394_2017_1443_MOESM2_ESM.docx]

Supplementary material for: Aune D, Sen A, Leitzmann MF, Norat T, Tonstad S, Vatten LJ. Body mass index and physical activity and the risk of diverticular disease: a systematic review and meta-analysis of prospective studies. Eur J Nutr 2017 DOI: 10.1007/s00394-017-1443-x

Supplementary Table 1.List of excluded studies and exclusion reason

| Exclusion reason | Reference |
| --- | --- |
| Abstract | [1-7] |
| Case-control study | [8-15] |
| Case only study | [16-35] |
| Cross-sectional study | [36-49] |
| Not relevant exposure | [50-52] |
| Review | [53] |

Reference List

1. Humes DJ, West J, Fleming KM, Spiller RC (2009) A case control study of risk factors for perforated diverticular disease. Gastroenterology Conference: Digestive Disease Week, DDWMay

2. Strate LL, Parasa S, Arnold AM, Siscovick D (2010) Cardiovascular risk factors and diverticular bleeding. Gastrointestinal Endoscopy Conference: Digestive Disease WeekApril

3. Hjern F, Wolk A, Hakansson N (2011) Overweight, obesity and physical inactivity are associated with symptomatic colonic diverticular disease. Diseases of the Colon and Rectum Conference: Annual Meeting of the American Society of Colon and Rectal Surgeons, ACSRSMay

4. Kim KH, Kim HY, Shin WG, Kim SJ (2011) Visceral obesity is associated with increased risk for acute diverticulitis in young adults korean patients. Gastroenterology Conference: Digestive Disease Week, DDWMay

5. Humes DJ, Jarvholm B, Ludvigsson JF (2015) Body mass index and the risk of symptomatic diverticular disease: A Swedish population based cohort study. Gut Conference: 2nd Digestive Disorders Federation Conference, DDFJune

6. Farrar WD, Erickson MS, Enders F (2015) Metabolic syndrome factors and the risk of diverticulosis. Gastroenterology Conference: Digestive Disease WeekApril

7. Freckelton J, Holt DQ, Borsaru A, Moore GT (2016) The role of body composition in diverticular disease. Journal of Gastroenterology and Hepatology (Australia) Conference: Australian Gastroenterology WeekOctober

8. Sorser SA, Hazan TB, Piper M, Maas LC (2009) Obesity and complicated diverticular disease: is there an association? South Med J 102:350-353

9. Suzuki K, Uchiyama S, Imajyo K, Tomeno W, Sakai E, Yamada E, Tanida E, Akiyama T, Watanabe S, Endo H, Fujita K, Yoneda M, Takahashi H, Koide T, Tokoro C, Abe Y, Kawaguchi M, Gotoh E, Maeda S, Nakajima A, Inamori M (2012) Risk factors for colonic diverticular hemorrhage: Japanese multicenter study. Digestion 85:261-265

10. Kopylov U, Ben-Horin S, Lahat A, Segev S, Avidan B, Carter D (2012) Obesity, metabolic syndrome and the risk of development of colonic diverticulosis. Digestion 86:201-205

11. Yamada E, Ohkubo H, Higurashi T, Sakai E, Endo H, Takahashi H, Uchida E, Tanida E, Izumi N, Kanesaki A, Hata Y, Matsuura T, Fujisawa N, Komatsu K, Maeda S, Nakajima A (2013) Visceral obesity as a risk factor for left-sided diverticulitis in Japan: a multicenter retrospective study. Gut Liver 7:532-538

12. Schneider LV, Millet I, Boulay-Coletta I, Taourel P, Loriau J, Zins M (2016) Right colonic diverticulitis in Caucasians: presentation and outcomes versus left-sided disease. Abdom Radiol (NY)

13. Khorrami M, Azrak MF, Keilin S (2010) Diverticulosis: Clinical predictors. A retrospective case-control study. Gastroenterology Conference: Digestive Disease Week, DDWMay

14. Sugihara Y, Kudo SE, Miyachi H, Misawa M, Okoshi S, Okada H, Yamamoto K (2015) Analysis of Risk Factors for Colonic Diverticular Bleeding: A Matched Case-Control Study. Gut Liver

15. Wolf C (2012) Diverticulitis: the relationship between body mass index and disease location, recurrence, and complications. Gastroenterol Nurs 35:46-51

16. Dobbins C, DeFontgalland D, Duthie G, Wattchow DA (2006) The relationship of obesity to the complications of diverticular disease. Colorectal Dis 8:37-40

17. Kim JH, Cheon JH, Park S, Kim BC, Lee SK, Kim TI, Kim WH (2008) Relationship between disease location and age, obesity, and complications in Korean patients with acute diverticulitis: a comparison of clinical patterns with those of Western populations. Hepatogastroenterology 55:983-986

18. Malhotra A, Shah AM, Shakov R, Baddoura W (2010) Does BMI help to predict a complicated course in diverticulitis of the young? A retrospective analysis. Gastroenterology Conference: Digestive Disease Week, DDWMay

19. Jeong JH, Lee HL, Kim JO, Tae HJ, Jung SH, Lee KN, Jun DW, Lee OY, Yoon BC, Choi HS, Hahm JS, Song SY (2011) Correlation between complicated diverticulitis and visceral fat. J Korean Med Sci 26:1339-1343

20. Kim SY, Oh TH, Seo JY, Jeon TJ, Seo DD, Shin WC, Choi WC, Jeong MJ (2012) The clinical factors for predicting severe diverticulitis in Korea: a comparison with Western countries. Gut Liver 6:78-85

21. Bose KP, Khorshidi I, Southern WN, Brandt LJ (2013) The impact of ethnicity and obesity on the course of colonic diverticulitis. J Clin Gastroenterol 47:160-164

22. Nagata N, Sakamoto K, Arai T, Niikura R, Shimbo T, Shinozaki M, Aoki T, Sekine K, Okubo H, Watanabe K, Sakurai T, Yokoi C, Yanase M, Akiyama J, Noda M, Uemura N (2015) Visceral fat accumulation affects risk of colonic diverticular hemorrhage. Int J Colorectal Dis 30:1399-1406

23. Docimo J, Lee Y, Chatani P, Rogers AM, Lacqua FJ (2016) Visceral to subcutaneous fat ratio predicts acuity of diverticulitis. Surgical Endoscopy and Other Interventional Techniques Conference:March

24. Docimo S Jr, Lee Y, Chatani P, Rogers AM, Lacqua F (2016) Visceral to subcutaneous fat ratio predicts acuity of diverticulitis. Surg Endosc

25. Bailey MB, Davenport DL, Procter L, McKenzie S, Vargas HD (2013) Morbid obesity and diverticulitis: results from the ACS NSQIP dataset. J Am Coll Surg 217:874-880

26. Yamada E, Takahashi H, Ohkubo H, Higurashi T, Sakai E, Endo H, Yamada M, Maruyama Y, Gushimiyagi M, Yamamoto T, Manabe N, Haruma K, Nakajima A (2013) Underweight is a risk factor for complicated diverticulitis in male: Multicenter study in Japan. United European Gastroenterology Journal Conference: 21st United European Gastroenterology Week Berlin Germany Conference Start:October

27. Park NS, Jeen YT, Choi HS, Kim ES, Kim YJ, Keum B, Seo YS, Chun HJ, Lee HS, Um SH, Kim CD, Ryu HS (2013) Risk factors for severe diverticulitis in computed tomography-confirmed acute diverticulitis in Korea. Gut Liver 7:443-449

28. Lebwohl B, Cao Y, Neugut AI, Rundle A (2009) Risk factors for diverticulosis in the era of screening colonoscopy. Gastroenterology Conference: Digestive Disease Week, DDWMay

29. Shim SG, Lee OY, Ahn YW, Joo YW, Kim H, Lee KN, Lee HL, Yoon BC, Choi HS, Lee MH, Hahm JS (2010) The relationship between abdominal fat amount and the diverticular disease. Gastroenterology Conference: Digestive Disease Week, DDWMay

30. Kim SY, Oh T-H, Jeon TJ, Seo DD, Shin WC, Choi W-C (2011) Clinical factors for predicting severe diverticulitis in Korea: A single center study. Gastroenterology Conference: Digestive Disease Week, DDWMay

31. Kawatkar AA, Longstreth GF, Yen L, Chu L-H, Chen W, Hodgkins P, Erder MH, Iyer RL, Nichol MB (2012) Predictors of recurrence of acute diverticulitis in an incident diverticulitis cohort. Gastroenterology Conference: Digestive Diease WeekMay

32. Sharma P, Eglinton T, Frizelle F (2014) The influence of body mass index, medications and chronic illness on the disease course of acute diverticulitis. Colorectal Disease Conference: Tripartite Colorectal Meeting of the American Society of Colon and Rectal Surgeons, ASCRS; Association of Coloproctology of GB and Ireland; the Section of Coloproctology, Royal Society of Medicine; the Colon and Rectal SurgJuly

33. Manabe N, Haruma K, Nakajima A, Yoshino J, Takahashi S, Yamada M, Maruyama Y, Gushimiyagi M, Yamamoto T, Oyamada H (2012) Clinical characteristics and risk factors of acute diverticulitis with abscess in Japan-analysis from japanese multicenter large study cohort. Gastroenterology Conference: Digestive Diease WeekMay

34. Savu MK, Abourjaily G, Logue A, Mayoral J, Ledesma E, Perry WB, Eid K (2012) Obesity increases risk of complications of diverticular disease in a va patient population. Surgical Endoscopy and Other Interventional Techniques Conference:March

35. Jalil AA, Cronin R, Comianos M, Mann M (2013) Risk factors for diverticular bleeding and re-bleeding: A united states community-based hospital study. American Journal of Gastroenterology Conference: 78th Annual Scientific Meeting of the American College of Gastroenterology San Diego, CA United States Conference Start:October

36. Song JH, Kim YS, Lee JH, Ok KS, Ryu SH, Lee JH, Moon JS (2010) Clinical characteristics of colonic diverticulosis in Korea: a prospective study. Korean J Intern Med 25:140-146

37. Fujimoto AI, Hashimoto M, Hoteya S, Iizuka T, Ogawa O, Mitani T, Matsui A, Nakamura M, Kikuchi D, Yamashita S, Furuhata T, Yamada A, Igarashi Y, Kaise M (2012) Obesity and gastrointestinal disease. Gastrointestinal Endoscopy Conference: Digestive Diease WeekApril

38. Sharara AI, El-Halabi MM, Mansour NM, Malli A, Ghaith OA, Hashash JG, Maasri K, Soweid A, Barada K, Mourad FH, El ZL (2013) Alcohol consumption is a risk factor for colonic diverticulosis. J Clin Gastroenterol 47:420-425

39. Peery AF, Sandler RS, Ahnen DJ, Galanko JA, Holm AN, Shaukat A, Mott LA, Barry EL, Fried DA, Baron JA (2013) Constipation and a low-fiber diet are not associated with diverticulosis. Clin Gastroenterol Hepatol 11:1622-1627

40. Self RB, Birmingham CL, Elliott R, Zhang W, Thommasen HV (2005) The prevalence of overweight adults living in a rural and remote community. The Bella Coola Valley. Eat Weight Disord 10:133-138

41. Yamada M, Manabe N, Nakajima A, Mizuno M, Nakazawa A, Oyamada H, Gushimiyagi M, Kanai T, Haruma K (2013) Clinical characteristics and risk factors of colonic diverticulosis-analysis from Japanese multicenter prospective cross-sectional study-yasuhiko maruyama. Gastroenterology Conference: Digestive Disease WeekMay

42. Yamamichi N, Shimamoto T, Takahashi Y, Sakaguchi Y, Kakimoto H, Matsuda R, Kataoka Y, Saito I, Tsuji Y, Yakabi S, Takeuchi C, Minatsuki C, Niimi K, sada-Hirayama I, Nakayama C, Ono S, Kodashima S, Yamaguchi D, Fujishiro M, Yamaji Y, Wada R, Mitsushima T, Koike K (2015) Trend and risk factors of diverticulosis in Japan: age, gender, and lifestyle/metabolic-related factors may cooperatively affect on the colorectal diverticula formation. PLoS One 10:e0123688

43. Wang FW, Chuang HY, Tu MS, King TM, Wang JH, Hsu CW, Hsu PI, Chen WC (2015) Prevalence and risk factors of asymptomatic colorectal diverticulosis in Taiwan. BMC Gastroenterol 15:40

44. Nagata N, Sakamoto K, Arai T, Niikura R, Shimbo T, Shinozaki M, Aoki T, Sekine K, Okubo H, Watanabe K, Sakurai T, Yokoi C, Akiyama J, Yanase M, Noda M, Itoh T, Mizokami M, Uemura N (2015) Visceral abdominal obesity measured by computed tomography is associated with increased risk of colonic diverticulosis. Journal of Clinical Gastroenterology 49 (10) (pp 816-822),09

45. Peery AF, Barrett PR, Park D, Rogers AJ, Galanko JA, Martin CF, Sandler RS (2015) A High-Fiber Diet Does Not Protect Against Asymptomatic Diverticulosis. Gastroenterology (no pagination)

46. Peery AF, Martin C, Galanko J, Sandler R (2016) Obesity is a risk factor for colonic diverticulosis. Gastroenterology Conference: Digestive Disease WeekApril

47. Manabe N, Haruma K, Nakajima A, Yamada M, Maruyama Y, Gushimiyagi M, Yamamoto T (2015) Characteristics of Colonic Diverticulitis and Factors Associated With Complications: A Japanese Multicenter, Retrospective, Cross-Sectional Study. Dis Colon Rectum 58:1174-1181

48. Dore MP, Pes GM, Marras G, Soro S, Rocchi C, Loria MF, Bassotti G (2016) Risk factors associated with colonic diverticulosis among patients from a defined geographic area. Tech Coloproctol 20:177-183

49. Afonso M, Pinto J, Veloso R, Freitas T, Carvalho J, Fraga J (2010) Obesity and visceral fat in diverticular disease of the colon. American Journal of Gastroenterology Conference: 75th Annual Scientific Meeting of the American College of Gastroenterology San Antonio, TX United States Conference Start:October

50. Skoldberg F, Svensson T, Olen O, Hjern F, Schmidt PT, Ljung R (203) A population-based case-control study on statin exposure and risk of acute diverticular disease. Scandinavian Journal of Gastroenterology 51 (2) (pp01

51. Stott C, Jamshed S, Torosis M, Kamal A (2011) Epidemiology of right-sided diverticulosis among Asian Americans. Gastroenterology Conference: Digestive Disease Week, DDWMay

52. Okamoto T, Watabe H, Kobayashi Y, Kambe H, Yoshida S, Isomura Y, Yamada A, Sugimoto T, Hirata Y, Yamaji Y, Koike K (2011) Case control study on risk assessment of colonic diverticular bleeding. Gastroenterology Conference: Digestive Disease Week, DDWMay

53. Martin D (2011) Physical activity benefits and risks on the gastrointestinal system. South Med J 104:831-837

Supplementary Table 2: Relative risks and 95% CIs from nonlinear dose-response analysis of BMI and diverticular disease incidence and complications

|  | Diverticular disease incidence | Diverticulitis incidence | Diverticular disease complications (bleeding, perforation or abscess) |
| --- | --- | --- | --- |
| BMI | RR (95% CI) | RR (95% CI) | RR (95% CI) |
| 17.5 | 0.87 (0.96-0.79) | - | 1.43 (0.94-2.16) |
| 20.0 | 1.00 | 1.00 | 1.00 |
| 22.5 | 1.15 (1.07-1.23) | 1.06 (0.93-1.20) | 0.94 (0.78-1.14) |
| 25.0 | 1.31 (1.17-1.47) | 1.15 (0.93-1.43) | 1.03 (0.80-1.33) |
| 27.5 | 1.50 (1.31-1.71) | 1.30 (1.01-1.68) | 1.22 (0.94-1.58) |
| 30.0 | 1.71 (1.52-1.94) | 1.54 (1.21-1.95) | 1.49 (1.17-1.89) |
| 32.5 | 1.96 (1.77-2.18) | 1.90 (1.51-2.38) | 1.85 (1.49-2.29) |
| 35.0 | 2.26 (2.00-2.54) | 2.47 (1.76-3.46) | 2.30 (1.90-2.78) |
| 37.5 | 2.60 (2.11-3.21) | 3.41 (1.82-6.38) | 2.84 (2.37-3.42) |
| 40.0 | 3.01 (2.06-4.39) | 5.01 (1.73-14.53) | 3.49 (2.88-4.23) |
| p_nonlinearity_ | 0.22 | 0.25 | <0.0001 |
